# HLA-DM Focuses on Conformational Flexibility Around P1 Pocket to Catalyze Peptide Exchange

**DOI:** 10.3389/fimmu.2013.00336

**Published:** 2013-10-17

**Authors:** Liusong Yin, Lawrence J. Stern

**Affiliations:** ^1^Department of Pathology, University of Massachusetts Medical School, Worcester, MA, USA; ^2^Department of Biochemistry and Molecular Pharmacology, University of Massachusetts Medical School, Worcester, MA, USA

**Keywords:** antigen presentation, epitope selection, MHCII-peptide complex, HLA-DM susceptibility, hydrogen bonds, conformational heterogeneity

## Abstract

Peptides presented by major histocompatibility complex class II (MHCII) molecules to CD4+ T cells play a central role in the initiation of adaptive immunity. This antigen presentation process is characterized by the proteolytic cleavage of foreign and self proteins, and loading of the resultant peptides onto MHCII molecules. Loading and exchange of antigenic peptides is catalyzed by a non-classical MHCII molecule, HLA-DM. The impact of HLA-DM on epitope selection has been appreciated for a long time. However, the molecular mechanism by which HLA-DM mediates peptide exchange remains elusive. Here, we review recent efforts in elucidating how HLA-DM works, highlighted by two recently solved co-structures of HLA-DM bound to HLA-DO (a natural inhibitor of HLA-DM), or to HLA-DR1 (a common MHCII). In light of these efforts, a model for HLA-DM action in which HLA-DM utilizes conformational flexibility around the P1 pocket of the MHCII-peptide complex to catalyze peptide exchange is proposed.

## Introduction

Major histocompatibility complex class II (MHCII) proteins are expressed constitutively on the surface of professional antigen-presenting cells, and induced by inflammatory stimuli on many other cell types. They display peptides derived from self or foreign antigens for recognition by CD4+ T cells to initiate and regulate many aspects of adaptive immunity. Newly synthesized MHCII molecules associate with the invariant chain chaperone, which directs nascent MHCII to endosomal compartments before degradation by endosomal-resident cathepsin proteases, leaving small peptide fragments known as CLIP (class II-associated invariant chain peptide) bound in the MHCII-peptide binding groove (1–7). The release of CLIP and subsequent loading of antigenic peptides onto MHCII is catalyzed by a non-classical MHCII molecule, HLA-DM (8). HLA-DM also serves as a peptide editor by mediating the exchange of bound antigenic peptides, selecting for presentation of peptides with higher kinetic stability (9).

HLA-DM-mediated peptide exchange has been shown to play a key role in epitope selection. It has been found that HLA-DM extinguishes the presentation of cryptic epitopes and stimulates the presentation of immunodominant epitopes (10), and that the effects of HLA-DM editing can be altered by manipulation of the kinetic stability of MHCII-peptide complexes (11). We and others have demonstrated that the kinetic stability of MHCII-peptide complexes in the presence of HLA-DM directly correlates with immunogenicity (12–14). However, some immunodominant epitopes are found to have low affinities and low kinetic stabilities; notably most derive from self-antigens and are often associated in autoimmune diseases (15–18). In these cases, it is possible that self-peptides bound to MHCII alleles escaped HLA-DM editing due to downregulation, inhibition, or deficiency of HLA-DM in antigen presenting cells. This idea is supported by the observation that expression of HLA-DM decreased significantly in rheumatoid arthritis patients (19).

Due to the key role of HLA-DM in selection of both foreign pathogenic epitopes and autoimmune self-reactive epitopes, the mechanism of HLA-DM-mediated peptide exchange has been studied intensively. Here, we review the literature on efforts to determine how HLA-DM mediates peptide exchange and focus on two recent co-structures of HLA-DM in complex with MHCII binding partners.

## Involvement and Contribution of Hydrogen Bonds to HLA-DM Susceptibility

The hydrogen bond network between peptide backbone and MHCII main chain (α51–α53) or conserved MHCII side-chain (α62, α69, β61, β81, and β82) residues, together with peptide side-chain binding P1, P4, P6, and P9 pockets are a characteristic feature of MHCII-peptide interaction (20, 21). The ability of HLA-DM to catalyze the exchange of a wide variety of peptides implicated the hydrogen bond network as an ideal target for HLA-DM (22). This idea was experimentally demonstrated in several studies, which in general highlighted a role for MHCII-peptide hydrogen bonds near P1 pocket (23–25). A seeming discrepancy came from two subsequent studies demonstrating that HLA-DM functions normally on MHCII-peptide complexes lacking conserved side-chain hydrogen bonds (26, 27), but it is important to note that neither study looked at the contribution of MHC main chain hydrogen bonds as described in Stratikos et al. Nevertheless, these studies suggested that individual hydrogen bonds may not be the key target for HLA-DM. A study of a MHCII mutant protein highly susceptible to HLA-DM action revealed weakened MHC-peptide hydrogen bonding, as shown by a novel hydrogen-deuterium exchange mass spectrometry assay, and structural alterations (discussed below), suggesting a connection between conformational features of MHCII-peptide complex, the MHCII-peptide hydrogen bond network, and HLA-DM susceptibility (28).

## Conformational Heterogeneity of MHCII-Peptide Complexes in HLA-DM Susceptibility

Peptide-free MHCII molecules and certain MHCII variants loaded with peptides can adopt conformations distinct from the canonical structure observed by X-ray crystallography as judged by hydrodynamic, spectroscopic, and electrophoretic criteria (29–34). A role for these conformational changes in determining HLA-DM susceptibility has been appreciated more recently (28, 35–39). A recent crystal structure of a MHCII variant with increased HLA-DM affinity revealed conformational lability in the alpha 3_10_ helical and extended region near the N-terminal end of the bound peptide (28). Similar but smaller changes can be observed in crystal structures of certain MHCII-peptide complexes (34). A model for HLA-DM-mediated peptide exchange was presented, in which transient or low-abundant MHCII-peptide conformers are targeted by HLA-DM.

## Insight from Recent HLA-DM-HLA-DO and HLA-DM-HLA-DR1 Structures

**Figure 1:**
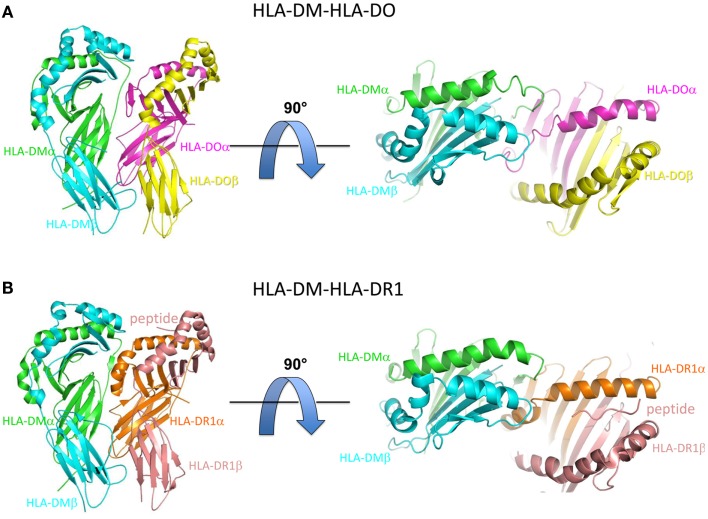
**Overview of HLA-DM-HLA-DO and HLA-DM-HLA-DR1 structures**. **(A)** Side view (left) and top view (right) of HLA-DM-HLA-DO structure (PDB ID: 4IOP). HLA-DMα is colored green, HLA-DMβ blue, HLA-DOα pink, and HLA-DOβ yellow. **(B)** Side view (left) and top view (right) of HLA-DM-HLA-DR1 structure (PDB ID: 4FQX). HLA-DM is colored the same, HLA-DR1α orange, and HLA-DR1β magenta. The peptide (colored magenta) is located in the C-terminal end of the peptide binding groove.

Recently, two X-ray crystal co-structures of HLA-DM bound to MHCII protein targets have helped to illuminate the mechanism of HLA-DM-mediated peptide exchange. In one, HLA-DM was bound to HLA-DO, a natural inhibitor of HLA-DM (40). In the other, HLA-DM was bound to HLA-DR1, a common MHCII allele, for which a truncated peptide was covalently trapped in the C-terminal side of the peptide binding site (41). In both cases HLA-DM binds to the MHCII protein in a side-by-side arrangement, with HLA-DM riding slightly above of the N-terminal side of the MHCII-peptide binding groove (Figure 1).

HLA-DO is a non-classical MHCII protein that forms a stable complex with HLA-DM and inhibits HLA-DM function, *in vitro* (42–44), and *in vivo* (45). In the HLA-DM-HLA-DO structure, HLA-DO adopts an overall topology highly similar to that of classical MHCII molecules such as HLA-DR1, with substantial alpha subunit alternations (40). The key differences are located in the 3_10_ helix and extended strand region of the alpha subunit, with HLA-DO residues αW43 and αF51 flipped out from their usual partially buried conformations to interact directly with HLA-DM. In addition, αF54 has moved into the P1 pocket, possibly giving insight into how empty MHCII is stabilized after peptide release (32). Given the fact that HLA-DM does not form stable complex with classical MHCII molecules, and the high structural similarity between HLA-DO and classical MHCII molecules, the HLA-DM-HLA-DO interactions may represent an intermediate stage during HLA-DM catalyzed peptide exchange from MHCII. This idea was supported by the almost identical match of HLA-DM and MHCII residues implicated by mutagenesis in the catalytic mechanism (33, 46, 47), and HLA-DM and HLA-DO residues found in the interface in the crystal structure (40).

**Figure 2:**
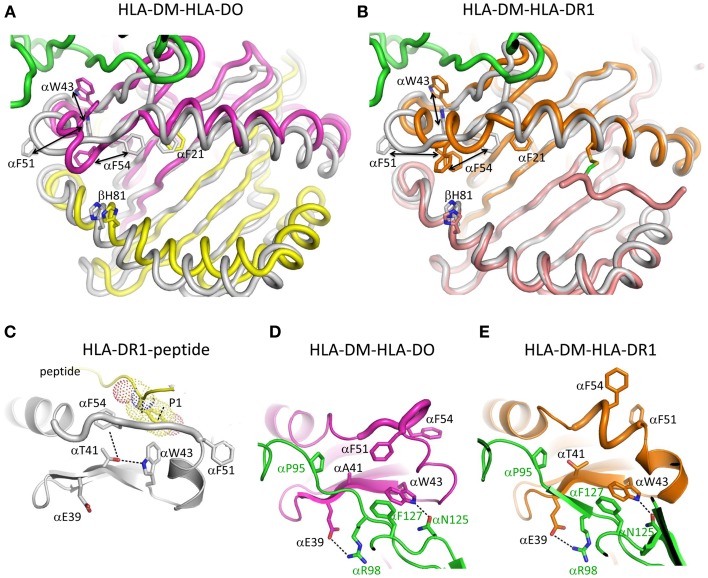
**Conformational changes in the alpha 310 helix and extended strand region**. **(A)** Overlay of HLA-DO from HLA-DM-HLA-DO structure with HLA-DR1-HA306-318 (PDB ID:1DLH). HLA-DMα is colored green, HLA-DOα pink, HLA-DOβ yellow, and HLA-DR1 gray. **(B)** Overlay of HLA-DR1 from HLA-DM-HLA-DR1 structure with HLA-DR1-HA306-318. HLA-DMα is colored green, HLA-DR1α, and HLA-DR1β from HLA-DM-HLA-DR1 orange and magenta, respectively, and HLA-DR1 from HLA-DR1-HA306-318 gray. The peptide (colored magenta) is covalently linked by a disulfide bond between a cysteine at peptide position P6 and HLA-DR1 αV65C. Orientations and movements of important aromatic residues in the vicinity of the P1 site are highlighted in **(A)** and **(B)**. Conformations of alpha 310 helix and extended strand region are shown for **(C)** HLA-DR1 bound with an influenza peptide (HA306-318), **(D)** HLA-DO bound with HLA-DM, and **(E)** HLA-DR1 bound with HLA-DM. Each subunit is colored the same as above. In **(C)** HLA-DR1 αW43 points into P1 pocket while αF51 and αF54 both point out; in **(D)** HLA-DO αW43 flips out to interact with αN125 from HLA-DM, while αF54 moves to the P1 pocket; in **(E)** HLA-DR1 αW43 also flips out to interact with αN125 from HLA-DM, while αF51 not αF54 moves to the P1 pocket. Other important residues are also indicated in each panel.

The structure of HLA-DM bound to HLA-DR1 carrying a covalently linked truncated peptide (41) also provides a model of an intermediate in HLA-DM-mediated peptide exchange. As in the HLA-DM-HLA-DO structure, αW43 in HLA-DR1 rotates out of the lateral wall of P1 pocket to directly interact with HLA-DM by forming a hydrogen bond with HLA-DM αN125. The αW43 flip is accompanied by a conformational change in MHCII alpha 3_10_ helix and extended strand region (α46–α55) adjacent to the P1 pocket. Although HLA-DO also exhibits conformational changes in this region when bound to HLA-DM, the conformation adopted is different (Figure 2). In addition, HLA-DR1 αF51 in the HLA-DM-HLA-DR1 structure, rather than HLA-DO αF54 in the HLA-DM-HLA-DO structure, repositions into the P1 pocket to stabilize empty MHCII. It is possible that the three views of the MHCII extended strand region observed in the various crystal structures represent different conformers from an ensemble adopted by a highly dynamic region.

Pos et al. suggested a model for HLA-DM-mediated peptide exchange based on the HLA-DM-HLA-DR1 structure. In that model, the N-terminus of a bound peptide dissociates from the P1 pocket, allowing HLA-DR1 αW43 to flip out and interact with HLA-DM. Meanwhile, HLA-DR1αF51 rotates into P1 pocket to stabilize partially empty HLA-DR1. An exchange peptide with a hydrophobic N-terminus could compete with HLA-DR1 αF51 for the binding to P1 pocket, and displace HLA-DM and the original peptide, reversing the conformational changes.

## Outstanding Questions in HLA-DM-Mediated Peptide Exchange

It is important to understand the process of HLA-DM-mediated peptide exchange, which is crucial for determining HLA-DM binding and susceptibility, and how peptide sequence relates to those properties. Proposed models agree on some aspects but differ in important ways on others. General agreement has been reached on the general structure of an intermediate in the peptide exchange reaction, in which HLA-DM is bound to MHCII in an altered conformation, involving a reorganized 3_10_ helix and extended strand region of the MHC II alpha subunit with disruption of MHCII-peptide hydrogen bonds, blockage of the P1 pocket, and inhibition of peptide binding. However, the disposition of aromatic residues in the P1 region, particularly αF51 and αF54, is not clear, and whether or not changes occur in the C-terminal side of peptide binding groove remains elusive. Also whether peptide is bound in the intermediate complex has not been tested directly and currently is not known.

The mechanism by which a HLA-DM-bound intermediate resolves to yield an exchanged MHCII-peptide complex also is not clear. One outstanding question is in whether the exchanging peptide binds dominantly via P1 pocket competition, or through interactions throughout the entire peptide binding groove. It is important to understand how peptide sequence determines the ability to be exchanged onto MHCII during antigen presentation, as this appears to be the crucial factor for epitope selection. A model involving a tetramolecular HLA-DM-MHCII-two peptide intermediate was proposed based on kinetics and spin-label studies (48). This model was suggested to explain observations of accelerated release of pre-bound peptide in the presence of free exchange peptide (49–51).

## Conclusion

Previous efforts and particularly two recent co-structures have shed light on the mechanism of HLA-DM-mediated peptide exchange. HLA-DM appears to promote peptide exchange by disrupting the MHCII-peptide hydrogen bond network at the N-terminal end of the peptide binding groove, and by disrupting MHCII-peptide interactions in the P1 pocket through stabilization of an altered conformation of the 3_10_ helix and extended strand region. Important questions still remain about how the sequence of MHCII-bound peptide determines MHCII-conformation, HLA-DM susceptibility, and peptide exchange function.

## Conflict of Interest Statement

The authors declare that the research was conducted in the absence of any commercial or financial relationships that could be construed as a potential conflict of interest.
